# Deep learning and clustering approaches for dental implant size classification based on periapical radiographs

**DOI:** 10.1038/s41598-023-42385-7

**Published:** 2023-10-06

**Authors:** Ji-Hyun Park, Hong Seok Moon, Hoi-In Jung, JaeJoon Hwang, Yoon-Ho Choi, Jong-Eun Kim

**Affiliations:** 1 Department of Prosthodontics, Yonsei University College of Dentistry, Yonsei-ro 50-1, Seodaemun-gu, Seoul, 03722 Korea; 2 Department of Preventive Dentistry and Public Oral Health, Yonsei University College of Dentistry, Seoul, 03722 Korea; 3 Department of Oral and Maxillofacial Radiology, School of Dentistry, Dental Research Institute, Pusan National University, Busan, 50612 Korea; 4 School of Computer Science and Engineering, Pusan National University, Busan, 46241 Korea

**Keywords:** Health care, Medical research

## Abstract

This study investigated two artificial intelligence (AI) methods for automatically classifying dental implant diameter and length based on periapical radiographs. The first method, deep learning (DL), involved utilizing the pre-trained VGG16 model and adjusting the fine-tuning degree to analyze image data obtained from periapical radiographs. The second method, clustering analysis, was accomplished by analyzing the implant-specific feature vector derived from three key points coordinates of the dental implant using the k-means++ algorithm and adjusting the weight of the feature vector. DL and clustering model classified dental implant size into nine groups. The performance metrics of AI models were accuracy, sensitivity, specificity, F1-score, positive predictive value, negative predictive value, and area under the receiver operating characteristic curve (AUC-ROC). The final DL model yielded performances above 0.994, 0.950, 0.994, 0.974, 0.952, 0.994, and 0.975, respectively, and the final clustering model yielded performances above 0.983, 0.900, 0.988, 0.923, 0.909, 0.988, and 0.947, respectively. When comparing the AI model before tuning and the final AI model, statistically significant performance improvements were observed in six out of nine groups for DL models and four out of nine groups for clustering models based on AUC-ROC. Two AI models showed reliable classification performances. For clinical applications, AI models require validation on various multicenter data.

## Introduction

The dental implant is a valuable treatment option for edentulous patients^1^. The long-term success rate and survival rate of dental implants are guaranteed, but mechanical and biological complications occur in patients with dental implants as time passes^2–4^. To manage various complications, detailed information on dental implants is essential.

In the case of mechanical complications, such as a fracture of dental prostheses, it is necessary to identify the dental implant systems. Afterward, the diameter of the implant should also be identified because the diameter of the dental implant determines the dimension of the connection between a superstructure and the dental implant. In the case of biological complications, especially peri-implantitis, radiographic measurement of bone level is a crucial factor^5–7^. In cases where previous radiographic examinations are unavailable, the diagnosis of peri-implantitis can be established when bone levels ≥ 3 mm apical to the most coronal aspect of the intra-osseous part of the implant are observed, accompanied by bleeding on probing^5^. Clinicians often measure bone loss using a relative ratio to the total implant length from the periapical radiograph. However, measuring exact bone loss or objectively comparing the rate of bone loss among patients with different implants is challenging using a relative bone loss ratio. A dental implant's length can serve as a reference metric in dental radiograph interpretation for radiographic measurement of bone level.

Numerous types of implants have emerged, so without medical records, getting specific and detailed information on dental implants is difficult^8^. In addition, after dental implant surgery, it is hard to observe or measure it directly, so periapical or panoramic radiographs are used to examine dental implants. However, identifying dental implants solely based on clinicians' experience can be time-consuming and costly without sufficient medical records.

The application of artificial intelligence (AI) in medical imaging analysis has expanded rapidly^9–14^. Also, in dentistry, AI models were used for the diagnosis of dental diseases such as dental caries or periodontal disease^15–17^. As a result, they improved the accuracy and reliability of diagnoses and aided dental professionals’ performances^17^. Active AI research was conducted to identify dental implant systems from dental radiography^18–24^. There were studies to obtain high performance in identifying dental implant types using small datasets, various deep convolutional neural networks (CNNs), and transfer learning. Recently a study showed that the deep learning (DL) model yielded accurate and valid results in identifying and classifying different dental implant systems with large-scale multicenter data^24^.

**Figure 1:**
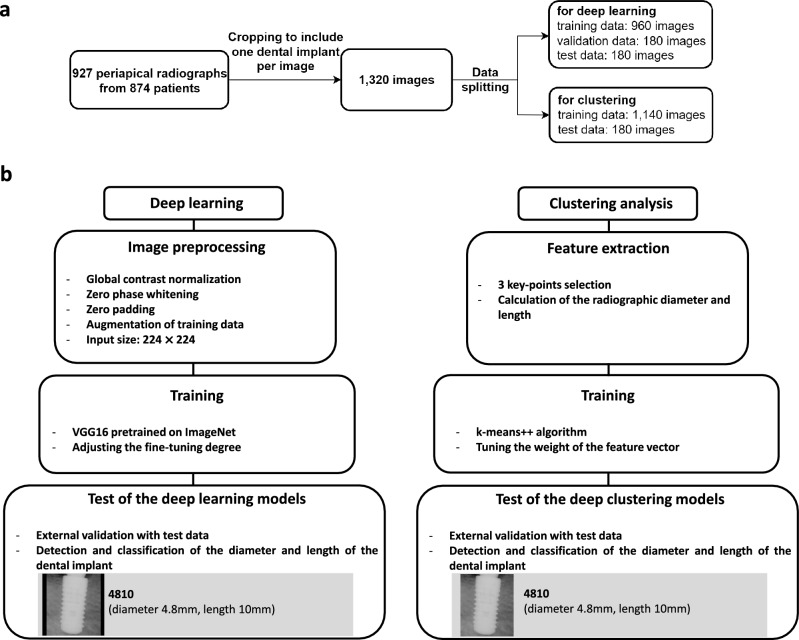
A schematic description of deep learning and clustering approaches: (**a**) data acquisition and data splitting for deep learning and clustering; (**b**) comparing of deep learning and clustering process.

However, few studies have investigated implant size, which plays an essential role in the production of implant prostheses and serves as a reference metric when interpreting dental radiographs. While recent research has focused on identifying dental implant systems, more investigations about automatically classifying implant size are needed. To address this research gap, this study aimed to develop and evaluate two automatic classification systems classifying the size of dental implants from periapical radiographs with DL and clustering (Fig. 1). It was hypothesized that the performance of the two AI models could be improved through the tuning process.

## Results

The label with a four-digit number represents the dental implant size, with the first two digits corresponding to the diameter and the last two digits corresponding to the length (3308, diameter 3.3 mm and length 8 mm; 3310, diameter 3.3 mm and length 10 mm; 3312, diameter 3.3 mm and length 12 mm; 4108, diameter 4.1 mm and length 8 mm; 4110, diameter 4.1 mm and length 10 mm; 4112, diameter 4.1 mm and length 12 mm; 4808, diameter 4.8 mm and length 8 mm; 4810, diameter 4.8 mm and length 10 mm; 4812, diameter 4.8 mm and length 12 mm).

### Classification of the implant size with DL

**Figure 2:**
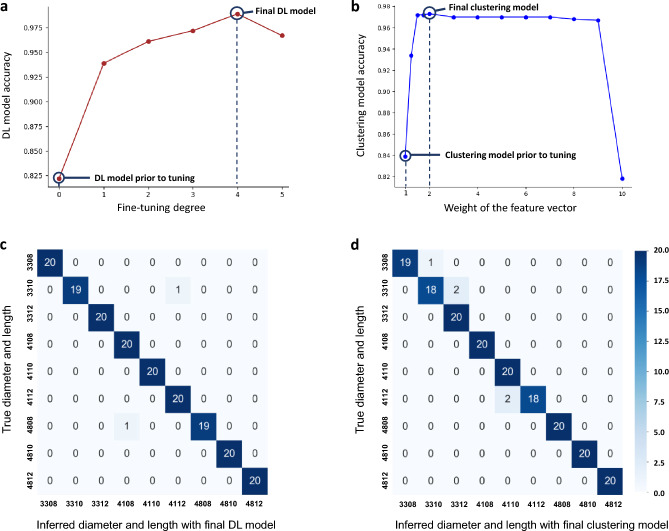
Results for implant size classification using deep learning and clustering approaches: (**a**) relationship between the fine-tuning degree and deep learning model accuracy; (**b**) relationship between the weight of the feature vector and clustering model accuracy; (**c**) confusion matrix of the final DL model result; (**d**) confusion matrix of the final clustering model result.

The final DL model with the best accuracy was chosen when the fine-tuning degree was four (Fig. 2a). Through adjusting the fine-tuning degree, the final DL model exhibited, in six out of nine groups, higher AUC-ROC values and statistically significant differences (*p* < 0.05) in terms of AUC-ROC compared to the model with the fine-tuning degree of zero.

Figure 2c shows a confusion matrix constructed by the final DL model with test data. Across the result of nine groups, the accuracy, sensitivity, specificity, F1-score, positive predictive value, negative predictive value, and AUC-ROC are above 0.994, 0.950, 0.994, 0.974, 0.952, 0.994, and 0.975 (95% CI, 0.926–1.00) respectively (Table 1). In the confusion matrix of the final DL model, there are two false predictions. One represents an inaccurate inference of the length and diameter, and the other shows an accurate inference of the length and an inaccurate inference of diameter. A confusion matrix and the performance evaluation results of the DL model with the fine-tuning degree of zero are shown (Supplementary Fig. S3 and Supplementary Table S4). Receiver operating characteristic (ROC) curve and area under the ROC curve (AUC-ROC) of the DL models with the best accuracy and the fine-tuning degree of zero in nine groups, and the results of the chi-square test are shown (Supplementary Fig. S5).

**Table 1:** Performance evaluation results of the final DL and clustering models.

Model	Label	TP	TN	FP	FN	ACC	SE	SP	F1-score	PPV	NPV	AUC-ROC (95% CI)
DL	3308	20	160	0	0	1.000	1.000	1.000	1.000	1.000	1.000	*1.000 (1.000–1.000)
	3310	19	160	0	1	0.994	0.950	1.000	0.974	1.000	0.994	*0.975 (0.926–1.000)
	3312	20	160	0	0	1.000	1.000	1.000	1.000	1.000	1.000	1.000 (1.000–1.000)
	4108	20	159	1	0	0.994	1.000	0.994	0.976	0.952	1.000	*0.997 (0.991–1.000)
	4110	20	160	0	0	1.000	1.000	1.000	1.000	1.000	1.000	*1.000 (1.000–1.000)
	4112	20	159	1	0	0.994	1.000	0.994	0.976	0.952	1.000	*0.997 (0.991–1.000)
	4808	19	160	0	1	0.994	0.950	1.000	0.974	1.000	0.994	0.975 (0.926–1.000)
	4810	20	160	0	0	1.000	1.000	1.000	1.000	1.000	1.000	1.00 (1.000–1.000)
	4812	20	160	0	0	1.000	1.000	1.000	1.000	1.000	1.000	*1.00 (1.000–1.000)
CL	3308	19	160	0	1	0.994	0.950	1.000	0.974	1.000	0.994	0.975 (0.926–1.00)
	3310	18	159	1	2	0.983	0.900	0.994	0.923	0.947	0.988	*0.947 (0.879–1.00)
	3312	20	158	2	0	0.989	1.000	0.988	0.952	0.909	1.000	0.994 (0.985–1.00)
	4108	20	160	0	0	1.000	1.000	1.000	1.000	1.000	1.000	1.000 (1.000–1.000)
	4110	20	158	2	0	0.989	1.000	0.988	0.952	0.909	1.000	*0.994 (0.985–1.000)
	4112	18	160	0	2	0.989	0.900	1.000	0.947	1.000	0.988	*0.950 (0.883–1.000)
	4808	20	160	0	0	1.000	1.000	1.000	1.000	1.000	1.000	1.000 (1.000–1.000)
	4810	20	160	0	0	1.000	1.000	1.000	1.000	1.000	1.000	1.000(1.000–1.000)
	4812	20	160	0	0	1.000	1.000	1.000	1.000	1.000	1.000	*1.000 (1.000–1.000)

*DL* deep learning, *CL* clustering, *TP* true positive, *TN* true negative, *FP* false positive, *FN* false negative, *ACC* accuracy, *SE* sensitivity, *SP* specificity, *PPV* positive predictive value, *NPV* negative predictive value, *AUC-ROC* area under the receiver operating characteristic curve, *CI* confidence interval.

The asterisk (*) indicates a statistically significant difference between the model performance before and after tuning, with a significance level of p < 0.05.

**Figure 3:**
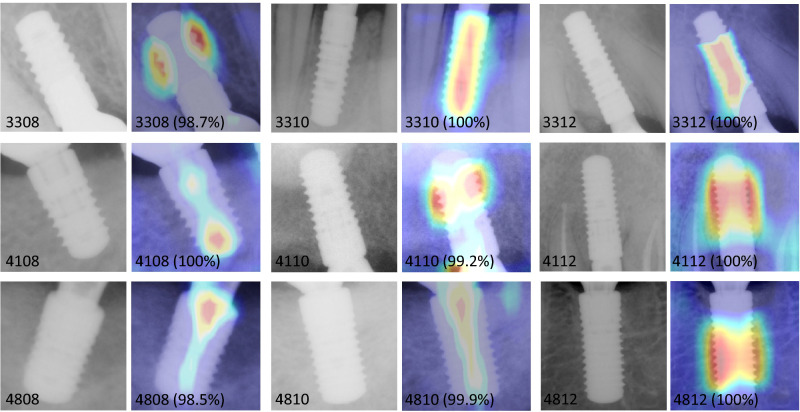
Bone level implant images and their Grad-CAM of the final deep learning model, described with true label, predicted label, and softmax value.

The input images of bone level implants and heat maps generated by gradient-weighted class activation mapping (Grad-CAM) were superimposed on the corresponding images, and they are presented in Fig. 3. In each heat map, red regions indicate higher activation values or importance, while blue regions represent lower activation values or importance in the prediction process^25^.

### Classification of the implant size with clustering

Using a test data set, external validation was conducted on the final clustering model with the best accuracy when the weight of the feature vector was two (Fig. 2b). Through adjusting the weight of the feature vector, the final clustering model exhibited, in four out of nine groups, higher AUC-ROC values and statistically significant differences (*p* < 0.05) in terms of AUC-ROC compared to the model with a weight of the feature vector set to one.

**Figure 4:**
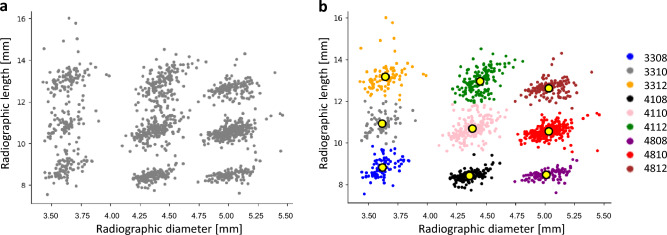
Scatter plot of the clustering analysis: (**a**) scatter plot of the feature vectors for clustering; (**b**) scatter plot of the final clustering model with the color code and representation of centroids of clusters (yellow circles).

Figure 2d shows a confusion matrix constructed by the final clustering model with test data. Across the result of nine groups, the accuracy, sensitivity, specificity, F1-score, positive predictive value,  negative predictive value, and AUC-ROC were above 0.983, 0.900, 0.988, 0.923, 0.909, 0.988, and 0.947 (95% CI, 0.879–1.00) respectively (Table 1). In the clustering model, each data point is assigned in each cluster based on Euclidean similarity, so the false predictions always appear on the cluster near the true predictions on the scatter plot of the feature vectors. In the confusion matrix of the final clustering model, the false prediction results represent accurate inferences on diameter and inaccurate inferences on length. The radiographic diameter and length are indicated on the *x*- and *y*-axes, respectively, on the scatter plot, and the cluster of each data is represented with color coding (Fig. 4). A confusion matrix and the performance evaluation results of the clustering model with the weight of the feature vector set to one are shown (Supplementary Fig. S6 and Supplementary Table S7). ROC curves and AUC-ROC values of the clustering models with the best accuracy and the weight of the feature vector set to one in nine groups and the results of the chi-square test are shown (Supplementary Fig. S8).

## Discussion

AI models in implant dentistry have the potential to recognize implant types, predict implant success, and optimize implant designs^26^. Many studies have attempted to develop methods for identifying the manufacturers and models of implants based on AI models^20–24^. Nevertheless, few studies have focused on the detailed diameter and length of dental implants in radiographic images. To the best of our knowledge, using deep learning or clustering to classify the size of dental implants has not yet been studied. The diameter of a dental implant plays an important role when selecting components for prosthetic restorations, while its length can serve as a reference for radiographic interpretations. Therefore, accurate information on the exact dimensions of the implant is essential for its long-term maintenance, including the management of mechanical and biological complications.

A study used a DL model to find six key points on periapical radiographs of dental implants and calculated the ratio of bone loss for the entire implant^27^. An automated key points detection system was proposed for calculating the percentage of bone loss to assess the severity of peri-implantitis. However, for the same ratio of the radiographic bone loss over the total implant length, the actual amount of bone loss varies proportionally with the implant length. Measuring bone loss amount rather than bone loss ratio is necessary for accurately interpreting radiographs for peri-implantitis diagnosis. Therefore, the present study aimed to develop automatic systems to identify the diameter and length of dental implants and to make it possible to measure the objective amount of bone loss.

In the clustering methods of this study, we employed not only three key points but also clustering analysis to infer the diameter and length of dental implants. In various prior studies of automatic lateral cephalogram analysis, deep learning was used for size measurements of various structures through landmark detection. In contrast, in this study, after extracting three key points, we utilized clustering analysis to infer the diameter and length of dental implants. This approach stems from differences in imaging conditions between lateral cephalograms and intraoral radiographs. In the case of lateral cephalograms, where the patient's sagittal plane is positioned parallel to the radiographic plate during imaging, the derived values can contribute to cephalogram analysis. However, from periapical radiographs of implants captured within the oral cavity, direct calculation of the implant's diameter and length is complicated by factors such as magnification resulting from radiographic imaging and reduction caused by the angle between the implant and the digital sensor. Particularly, the angular issue between the object and the digital sensor introduces limitations based on the anatomical structures within the oral cavity, varying according to the inclination of implants not visible within  the bone and different dental regions such as incisors, premolars, and molars. This study explored the feasibility of transforming radiographic diameter and length, obtained through landmark usage, into actual implant diameter and length through classification, employing machine learning techniques, specifically clustering.

**Figure 5:**
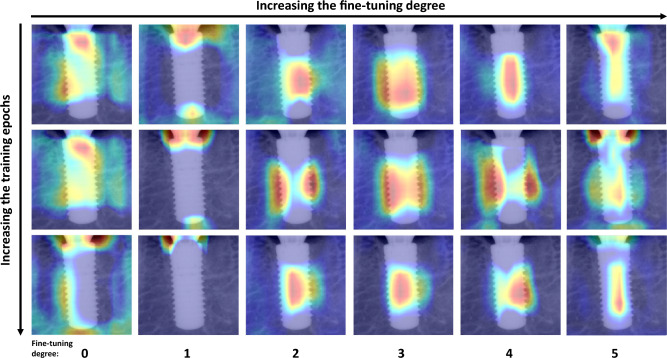
Grad-CAM images for different fine-tuning degrees and training epochs in deep learning approach.

In medical image analysis, AI models should be explainable to ensure the clinical relevance and reliability of the model's results for medical practitioners. Unfortunately, DL models often operate like black boxes, so various techniques, including Grad-CAM, were developed^25^. The range of layers to be fine-tuned is an important factor in achieving optimal performance on a new task in transfer learning. Figure 5 shows the Grad-CAM images with various freezing ranges and training epochs. When the fine-tuning degree of a DL model is zero or one, there is a tendency for the heatmap to appear in the background or outside the implant area. As the fine-tuning degree increases, there is a tendency for the heat map to concentrate and appear on the implant area. However, training a CNN architecture with only a small amount of data can result in overfitting and limited generalization. Therefore, when applying a DL model to limited data, it is crucial to perform appropriate fine-tuning^28^.

In applying a clustering analysis, a feature vector composed of the radiographic diameter and length of the dental implant was used in this study. All images were visualized in the scatter plot of the feature vectors, so it was possible to interpret how closely the feature vector of the dental implant image was positioned to the centroid or if it was positioned between two different clusters. This scatter plot serves as a map-like guide for dividing implant groups, assisting in the explainability of the clustering model.

In the case of using deep learning methods for bone level implants, there were two false predictions. In these cases, either the length and diameter were incorrectly predicted to be larger, or the length was predicted correctly, but the diameter was incorrectly predicted. In the case of using clustering methods for bone level implants, there were five false predictions. The diameter was predicted correctly in these cases, but the length was predicted incorrectly. The clustering model exhibited reliable results in inferring the diameter. If this trend is similarly observed across a wider range of implant systems and more datasets on external validation, there is a possibility that using both the DL model and clustering model in tandem for analyzing a single implant image could serve complementary roles. In cases where the predicted diameter and length of an implant differ between the two models, it is conceivable to give precedence to the predictions of the clustering model, which is speculated to be more reliable for diameter prediction. Subsequently, confirming the vector corresponding to the radiographic diameter and length of that image at the position of the feature map, and then comparing the predicted lengths from both the DL model and clustering model, while simultaneously considering distortions caused by the image's radiographic angulation, would facilitate a more rational decision-making process.

In the study, the performances of two AI models were improved through the tuning process. The fine-tuning degree was chosen as four through external validation with test data in the DL approach. When comparing the results of the tuning process before and after, based on the AUC-ROC values for the test dataset, a statistically significant performance improvement was observed in six out of the nine groups after tuning, when the tuning degree was set to four. In the clustering approach, comparing the results of the weight of the feature vector set to one and two, based on the AUC-ROC values for the test dataset, four of the nine groups demonstrated a statistically significant improvement in performance after tuning. As far as we know, no research has been conducted on utilizing deep learning or clustering approaches to classify the sizes of dental implants. Therefore, we could not compare the performance of our study's results with those of other studies.

This study had limitations. First, we utilized a limited set of images from periapical radiographs with Straumann bone level implants. Due to the limitations in data availability, our study utilized only periapical radiographs of a limited dataset of Straumann bone level implants to classify dental implant sizes using deep learning and clustering methods. Subsequent research should encompass more diverse dental implant systems and account for complex clinical scenarios to evaluate the performance of these AI models. For generalizability, the model should be trained on a large and diverse dataset that adequately represents the variations and complexities of the target problem^29^. Recently, a study identified 25 different systems of dental implants using 37,442 periapical and 113,291 panoramic images^24^. Also, for enhancing the reliability and reproducibility of AI models detecting and classifying the diameter and length of various dental implants, further investigation is crucial to establish a comprehensive, large-scale dataset including various dental implant systems and diameters and lengths of dental implants. Second, the dataset included only a periapical modality. In clinical practice, the limited information may include radiographs from different modalities, such as panoramic radiographs. Therefore, when constructing a dataset and conducting further studies, it is necessary to expand the scope by including radiographs from other modalities. Third, to evaluate the improvement in model performance, we compared the results before and after the tuning process for each AI approach^30,31^. For clinical validity assessment, it is necessary to compare the performance of dental professionals across multiple groups based on their experience, or it would be necessary to investigate how much these automated systems can enhance the decision performance of dental professionals. In previous studies focusing on classifying dental implant systems, they presented the results comparing the performance of AI models and dental professionals. In many of these studies, experts often conducted performance evaluations on human experts assuming prior knowledge in distinguishing dental implant systems. Dental professionals are skilled at distinguishing dental implant systems. However, progressing beyond implant system differentiation, distinguishing the diameter and length of these implants requires even more prior knowledge. Therefore, presenting human experts with a comparable quantity of training images annotated with dental implant sizes, akin to AI models, and accurately and efficiently evaluating the extent to which their performance improves, is imperative. Fourth, training models with imbalanced data can cause bias and performance deterioration in minority groups^32^. The model tends to prioritize the majority class due to its higher prevalence, reducing performance for the underrepresented classes. To overcome these limitations, future studies should aim to collect additional data from minority groups. Additionally, utilizing synthesized data from suitable generative models could effectively address these limitations^33,34^. Fifth, it will be essential to conduct tests to determine which scenarios deal with various dental implant systems and sizes with various AI approaches to find more effective methods. The results of this paper discussed the model performance of the fine-tuned VGG16. While our initial testing of ResNet50 and InceptionV3 in the study yielded performance lower than that of VGG16, they still exhibited reliable performance. ResNet50 and InceptionV3 demonstrated consistent performance not only in our study but also across various other research studies. Future research is needed to focus on a wider range of architectures, highlighting deep learning using various CNN architectures, such as DenseNet, MobileNet, EfficientNet, Xception, ResNeXt, SENet, and RegNet.

The AI models developed in this study have limitations in detecting various dental implant systems and different diameters and lengths. However, this research went beyond previous studies that focused on classifying implant systems and aimed to provide more detailed information about dental implants from periapical radiographs. By further advancing this approach, it has the potential to efficiently manage patients with dental implants in a clinical setting and offer an objective metric reference for dental radiograph interpretation.

## Conclusion

Automatic classification of the size of bone level implants can be achieved through DL and clustering. The performances of two AI models were improved through the tuning process. DL involves obtaining features through a training process with transfer learning and fine-tuning. On the other hand, the clustering model was developed by selecting an appropriate feature using three key points and tuning the weight of the feature vector. As a result, they can improve the efficiency and accuracy of implant assessment, assist dental professionals in making informed decisions, and enhance patient outcomes in dental implant treatments.

## Materials and methods

### Ethics

The Institutional Review Board (IRB) of Yonsei University Dental Hospital approved this study (Approval number: 2-2022-0067). The IRB of Yonsei University Dental Hospital waived the requirement to obtain individual informed consent, so no participants were provided written or verbal informed consent since this study had a noninterventional retrospective design, and all data were evaluated anonymously. All methods in this study were performed in accordance with the relevant guidelines and regulations.

### Data acquisition and data splitting

This study focused on the Straumann bone level implant (Institut Straumann, Basel, Switzerland). Periapical radiographs of Straumann bone level implants were included based on the inclusion criteria, which allowed for verification of diameter and length from the electronic medical records of dental implant first surgery. To replicate diverse clinical scenarios, the included periapical radiographs contained loaded implants, implants with healing abutments, and implants with cover screws. Cases, where dental implant images exhibited significant blurring or distortion due to movement during imaging or unusual imaging angles, were excluded from the study. We collected 927 periapical radiographs obtained from 874 patients aged 19–85 who underwent periapical radiography using the paralleling technique with 60 kVp, 7 mA, and 0.08–0.1 s between 2005 and 2022.

All periapical radiographs were cropped to display one dental implant per image. After cropping 927 periapical radiographs, 1320 images containing bone level implants were obtained (Fig. 1a). Each image was annotated with a four-digit number representing the dental implant's diameter and length, with the first two digits corresponding to the diameter and the last two corresponding to the length. A board-certified prosthodontist performed the process. Subsequently, cropped images underwent a thorough verification process. This involved cross-referencing each image with the corresponding electronic medical record entry. Initially, the process encompassed labeling to ascertain the number of threads, the characteristic appearance of the implant apex, as well as the proportional representation of diameter and length on the image itself. This comprehensive approach ensured the refinement of erroneously annotated data. The resulting process yielded a meticulously curated ground truth dataset. For the DL process, to prevent class imbalance, the validation dataset and testing dataset were structured with an equal distribution of 20 data points per class, while the remaining data was designated as the training dataset. The entire 1320 images were divided into 960 images for training, 180 images for validation, and 180 images for testing (Supplementary Table S1). For the clustering process, the entire 1320 images were divided into 1140 images for training and 180 images for testing (Supplementary Table S2). The testing dataset used for the performance evaluation of both AI models consisted of the same set of 180 images. The study was conducted according to the checklist regarding AI in dental research^31^.

### DL approach for bone level implant size classification

The cropped images were preprocessed with global contrast normalization and zero-phase whitening. The diversity of the training data was increased by applying various transformations to the images. The training data was randomly augmented using horizontal flipping, vertical flipping, rotation (range of ± 10°), width shifting (range of ± 0.1), height shifting (range of ± 0.04), zooming (range of ± 0.02), and shear (range of ± 0.01). As a result, the training data has been augmented by 16 times. The input image size was 224 by 224.

**Figure 6:**
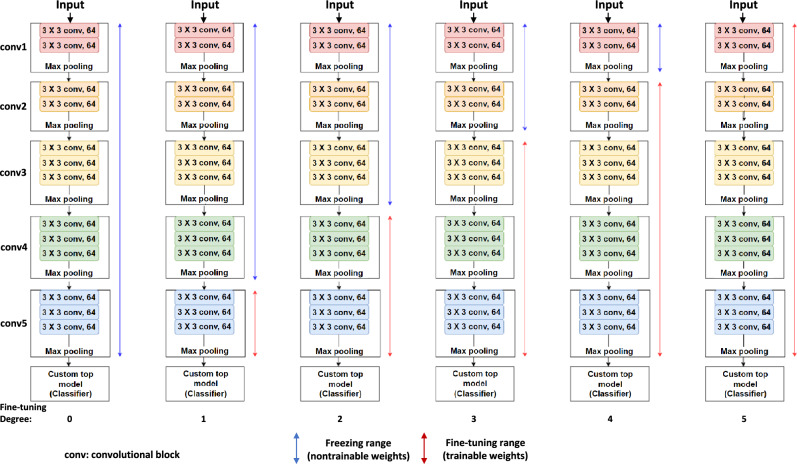
Six cases of the pre-trained VGG16 model by adjusting the fine-tuning degree.

VGG16, ResNet50, and InceptionV3 were tested as potential CNN architectures. The results showed accuracy values of 0.989, 0.961, and 0.967, respectively, with the test dataset, and VGG16 was chosen^35^. VGG16 model, pre-trained with ImageNet, was used for transfer learning^36^. VGG16 architecture comprises five convolutional blocks. In this study, the fine-tuning degree, in the range of zero to five, was defined as the number of blocks, which was trained with our training data, and the other convolutional blocks were frozen as the weight of ImageNet (Fig. 6). External validation was performed on six fine-tuned VGG16 models using the test data. The model achieving the best accuracy was chosen for the final DL model^37^.

Early stopping was used with the patience set to five to prevent overfitting. The learning rate was 0.00002 using the Adam optimizer. The batch size was set to 16. Dropout regularization with a rate of 0.5 was applied to prevent overfitting. The DL models were trained for 15 epochs with a possibility of early stopping and developed in Python 3.8.16 and TensorFlow 2.9.2 under a single NVIDIA RTX 3090.

### Clustering approach for bone level implant size classification

**Figure 7:**
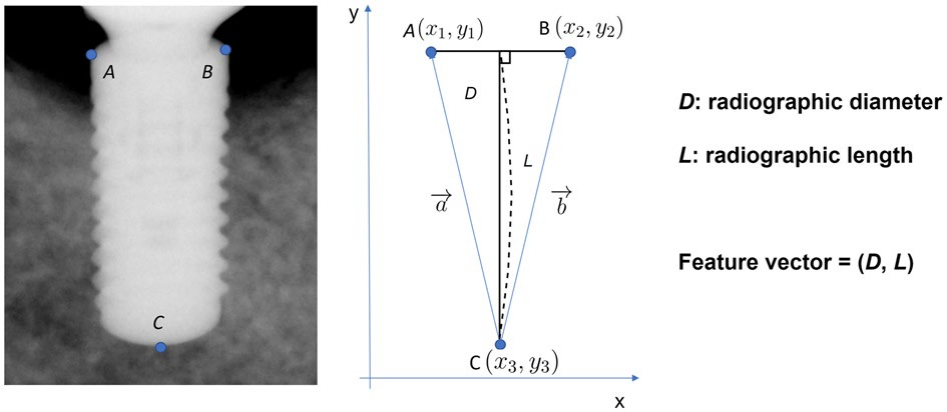
Key point selection and feature extraction for clustering.

Three key points (points A, B, and C) were labeled on cropped bone level implant images and used to calculate the radiographic diameter and length (Fig. 7). In this study, the diameter of the dental implant is the implant body, except for thread depth. $\overline{AB}$ corresponds to the diameter of the dental implant on a periapical radiograph, and point C corresponds to the dental implant apex. Key point annotation was performed using the annotation tool LabelMe (MIT Computer Science and Artificial Intelligence Laboratory, retrieved from https://github.com/wkentaro/labelme). The type of annotation was a polygon. The *x*- and *y*-coordinates of the three key points were extracted from a JSON format file. *D* means radiographic diameter, *L* means radiographic length, and *S* means the area of triangle ABC yields (Eqs. (1)–(5)).

1 $$D= \sqrt{{({x}_{2 }-{x}_{1})}^{2}+ {({y}_{2 }-{y}_{1})}^{2}}$$

2 $$S= \frac{1}{2}DL$$

3 $$S= \frac{1}{2} \left|\overrightarrow{a} \times \overrightarrow{b}\right|$$

4 $$S= \frac{1}{2} \left|{x}_{1}{y}_{2}+{x}_{2}{y}_{3}+{x}_{3}{y}_{1}- {x}_{2}{y}_{1}-{x}_{3}{y}_{2}-{x}_{1}{y}_{3}\right|$$

5 $$L= \frac{1}{D} \left|{x}_{1}{y}_{2}+{x}_{2}{y}_{3}+{x}_{3}{y}_{1}- {x}_{2}{y}_{1}-{x}_{3}{y}_{2}-{x}_{1}{y}_{3}\right|$$

Through the above equations, *D* and *L* were calculated in pixel units, so they were changed to the values in millimeters by multiplying themselves by the imager pixel spacing value, a DICOM metadata element, corresponding to the physical distance measured at the front plane of the image receptor housing between the centers of adjacent pixels. *D* and *L* were extracted from each image as a feature vector to be used for clustering.

The radiographic diameter and length indicated on the *x*- and *y*-axes, respectively, and the ground truth cluster of each data was represented with color coding. The three key points in each of the 1320 images of bone level implants were used to calculate the radiographic diameter and length for making a feature vector to train and test the clustering model.

This study used a type of *k*-means clustering called *k*-means++, which can improve the clustering process produced by the *k*-means algorithm by selecting centroids that are well-distributed throughout the data set^38,39^. The centroid represents the central point of a cluster. The centroid serves as a representative value or can be used to measure distances between clusters. As the number of clusters, *k* was set to nine because the study aimed to classify nine groups of the different diameters and lengths of the dental implants. A two-dimensional coordinate space was used with the radiographic diameter and length set as the *x*- and *y*-axes, respectively (Fig. 7). The study aimed to find the centroid for assigning clusters well to the ground-truth group from nine clusters by tuning the weight of the feature vector.

6 $$f : {R}^{2}\to {R}^{2},\quad where\, f\left(D,L\right)=\left(wD, L\right) \,\, and\,\, w>0$$

Function *f* denotes a linear transformation that modifies the diameter component of a vector by scaling with a factor *w* while keeping the vertical component unchanged (Eq. (6)). This transformation stretched or compressed the vector along the horizontal axis while keeping its length constant along the vertical axis.

The changes in the vector space according to the value of *w* and the resulting *k*-means clustering outcomes were evaluated. This research set *w* within the range of one to 10, and we adjusted it to improve the performance of the clustering model. The same computing resources used to develop the DL model were also employed to develop the clustering model.

### Statistical analysis and model performance evaluation

Statistical analysis was performed with the Python sklearn library and Stata software (StataCorp, College Station, TX) version 18. With the results of the two final models with DL and clustering approaches, the accuracy, sensitivity, specificity, F1-score, positive predictive value (PPV), negative predictive value (NPV), and AUC-ROC were calculated based on the confusion matrix (Eqs. (7)–(12), TP: true positive, TN: true negative, FP: false positive, FN: false negative, PPV: positive predictive value, NPV: negative predictive value. The recall is also known as sensitivity, and the precision is also known as PPV).

7 $$Accuracy=\frac{TP+TN}{TP+TN+FP+FN}$$

8 $$Sensitivity=\frac{TP}{TP+FN}$$

9 $$Specificity=\frac{TN}{TN+FP}$$

10 $$F1-score=\frac{2*Recall*Precision}{Recall+Precision}$$

11 $$PPV=\frac{TP}{TP+FP}$$

12 $$NPV=\frac{TN}{TN+FN}$$

Comparisons of performances were conducted in each two AI approaches between the model with the best accuracy and the model before tuning process. The chi-square test was used as a statistical test, and the statistical significance level was set to *p* = 0.05.

### Supplementary Information

Supplementary Information.

## Data Availability

The data sets generated or analyzed during the current study are not publicly available in order to preserve patient confidentiality but are available from the corresponding authors on reasonable request.
