# Supplementary material for: Deep learning and clustering approaches for dental implant size classification based on periapical radiographs
Source: Sci Rep. 2023 Oct 6;13:16856. doi: 10.1038/s41598-023-42385-7 (PMC10558577; doi:10.1038/s41598-023-42385-7)
Supplement: Supplementary file 1 — Supplementary Information. [file 41598_2023_42385_MOESM1_ESM.pdf]

Deep learning and clustering approaches for dental implant size classification based on periapical radiographs

Ji-Hyun Park[1], Hong Seok Moon[1], Hoi-In Jung[2], JaeJoon Hwang[3], Yoon-Ho Choi[4], and Jong-Eun Kim[1]

Supplementary Table S1. Bone level implant image data splitting and composition for the deep learning approaches

Implant size	Entire data	Training data	Validation data	Test data
3308	91	51	20	20
3310	80	40	20	20
3312	95	55	20	20
4108	144	104	20	20
4110	204	164	20	20
4112	157	117	20	20
4808	120	80	20	20
4810	275	235	20	20
4812	154	114	20	20
Total	1320	960	180	180

Supplementary Table S2. Bone level implant image data splitting and composition for the clustering approaches.

Implant size	Entire data	Training data	Test data
3308	91	71	20
3310	80	60	20
3312	95	75	20
4108	144	124	20
4110	204	184	20
4112	157	137	20
4808	120	100	20
4810	275	255	20
4812	154	134	20
Total	1320	1140	180

Supplementary Figure S3. Confusion matrix of the deep learning model with the fine-tuning degree set to zero.

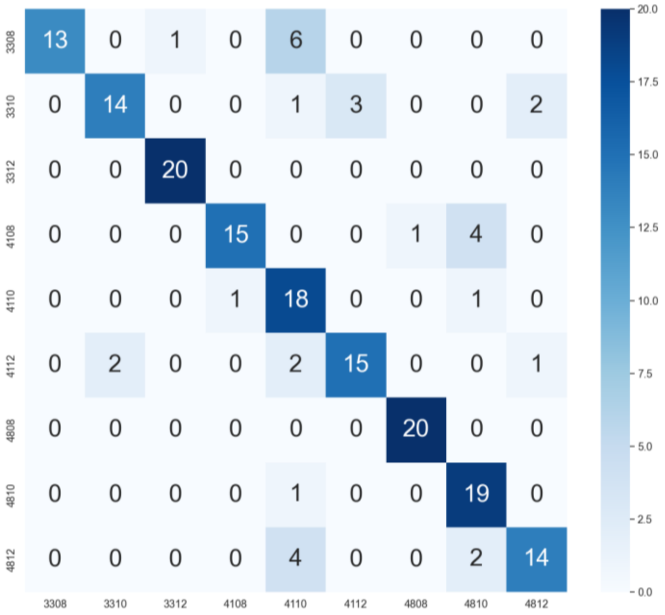

Supplementary Table S4. Performance evaluation results of the deep learning model with the fine-tuning degree set to zero.

Model	Label	TP	TN	FP	FN	ACC	SE	SP	F1-score	PPV	NPV	AUC-ROC (95% CI)
DL	3308	13	160	0	7	0.961	0.650	1.00	0.788	1.00	0.958	*0.825 (0.718-0.932)
	3310	14	158	2	6	0.956	0.700	0.988	0.778	0.875	0.963	*0.844 (0.740-0.947
	3312	20	159	1	0	0.994	1.00	0.994	0.976	0.952	1.00	0.997 (0.991-1.00)
	4108	15	159	1	5	0.967	0.705	0.994	0.833	0.938	0.97	*0.872 (0.774-0.969)
	4110	18	146	14	2	0.911	0.900	0.913	0.692	0.563	0.986	*0.906 (0.835-0.977)
	4112	15	157	3	5	0.956	0.750	0.981	0.789	0.833	0.969	*0.866 (0.768-0.964)
	4808	20	159	1	0	0.994	1.00	0.994	0.976	0.952	1.00	0.997 (0.991-1.00)
	4810	19	153	7	1	0.956	0.950	0.956	0.826	0.730	0.994	0.953 (0.902-1.00)
	4812	14	157	3	6	0.950	0.700	0.981	0.757	0.824	0.963	*0.841 (0.737-0.944)

DL, deep learning; TP, true positive; TN, true negative; FP, false positive; FN, false negative; ACC, accuracy; SE, sensitivity; SP, specificity; PPV, positive predictive value; NPV, negative predictive value; AUC, area under the receiver operating characteristic curve; CI, confidence interval. The asterisks (*) indicate statistically significant differences between the model performance before and after tuning, with a significance level of $p < 0.05$.

Supplementary Figure S5. ROC curves and AUC values of the deep learning models with the best accuracy and the fine-tuning degree set to zero in each nine groups

(a) ROC curves and AUCs on 3308 group (diameter 3.3mm, length 8mm). dl_1: the final DL model, dl_1_old: the DL model with the fine-tuning degree set to zero.

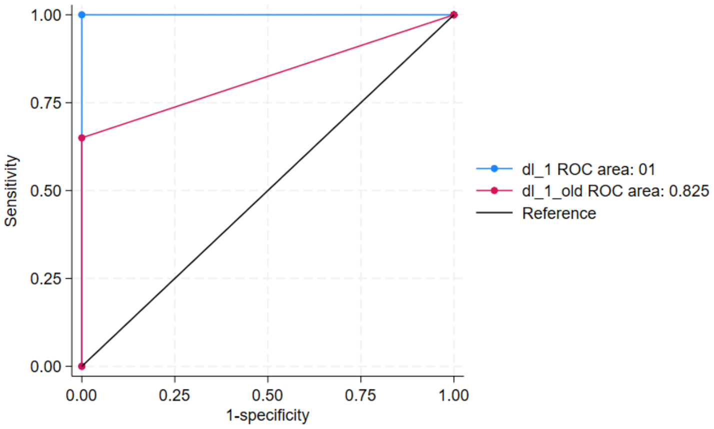

	Obs	ROC area	Std. err.	Asymptotic normal [95% conf.	interval]
dl_1	180	1.0000	0.0000	1.00000	1.00000
dl_1_old	180	0.8250	0.0547	0.71777	0.93223

H0: area(dl_1) = area(dl_1_old)
chi2(1) = 10.23 Prob>chi2 = 0.0014

(b) ROC curves and AUCs on 3310 group (diameter 3.3mm, length 10mm). dl_2: the final DL model, dl_2_old: the DL model with the fine-tuning degree set to zero.

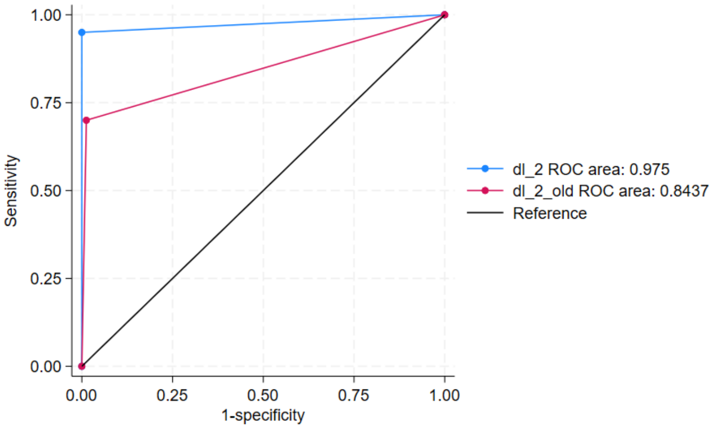

	Obs	ROC area	Std. err.	Asymptotic normal [95% conf.	interval]
dl_2	180	0.9750	0.0250	0.92600	1.00000
dl_2_old	180	0.8437	0.0528	0.74036	0.94714

H0: area(dl_2) = area(dl_2_old)
chi2(1) = 6.93 Prob>chi2 = 0.0085

(c) ROC curves and AUCs on 3312 group (diameter 3.3mm, length 12mm). dl_3: the final DL model, dl_3_old: the DL model with the fine-tuning degree set to zero.

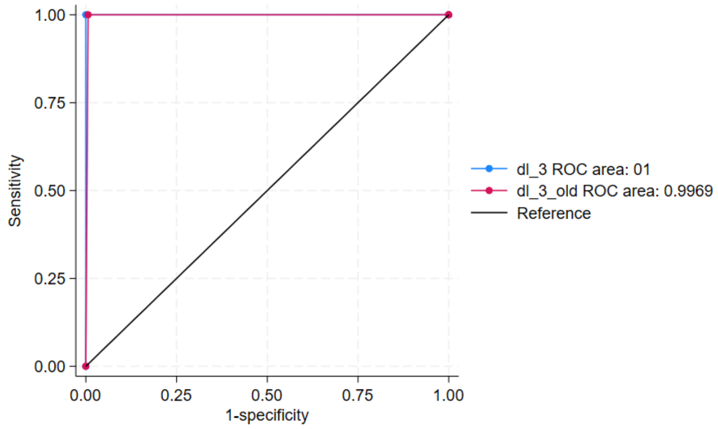

	Obs	ROC area	Std. err.	Asymptotic normal [95% conf.	interval]
dl_3	180	1.0000	0.0000	1.00000	1.00000
dl_3_old	180	0.9969	0.0031	0.99075	1.00000

H0: area(dl_3) = area(dl_3_old)
chi2(1) = 1.00 Prob>chi2 = 0.3173

(d) ROC curves and AUCs on 4108 group (diameter 4.1mm, length 8mm). dl_4: the final DL model, dl_4_old: the DL model with the fine-tuning degree set to zero.

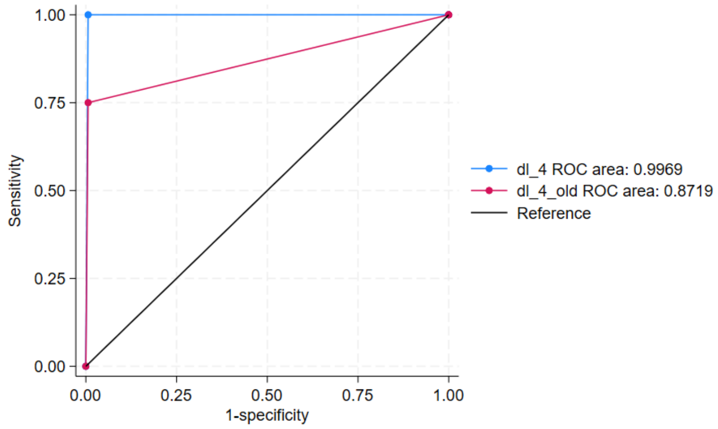

	Obs	ROC area	Std. err.	Asymptotic normal [95% conf.	interval]
dl_4	180	0.9969	0.0031	0.99075	1.00000
dl_4_old	180	0.8719	0.0498	0.77433	0.96942

H0: area(dl_4) = area(dl_4_old)
chi2(1) = 6.28 Prob>chi2 = 0.0122

(d) ROC curves and AUCs on 4110 group (diameter 4.1mm, length 10mm). dl_5: the final DL model, dl_5_old: the DL model with the fine-tuning degree set to zero.

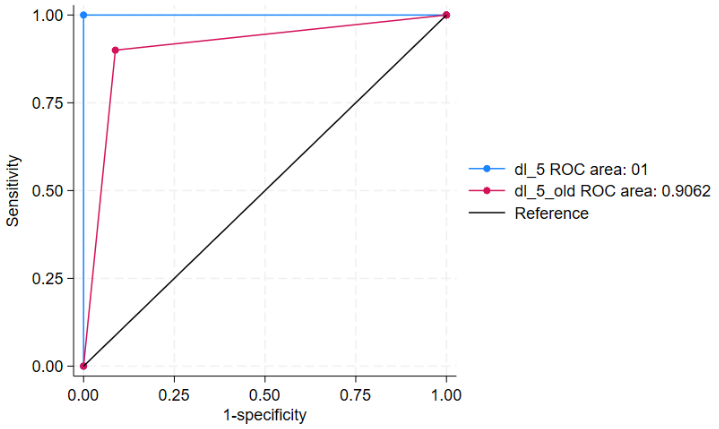

	Obs	ROC area	Std. err.	Asymptotic normal [95% conf.	interval]
dl_5	180	1.0000	0.0000	1.00000	1.00000
dl_5_old	180	0.9062	0.0362	0.83532	0.97718

H0: area(dl_5) = area(dl_5_old)
chi2(1) = 6.71 Prob>chi2 = 0.0096

(e) ROC curves and AUCs on 4112 group (diameter 4.1mm, length 12mm). dl_6: the final DL model, dl_6_old: the DL model with the fine-tuning degree set to zero.

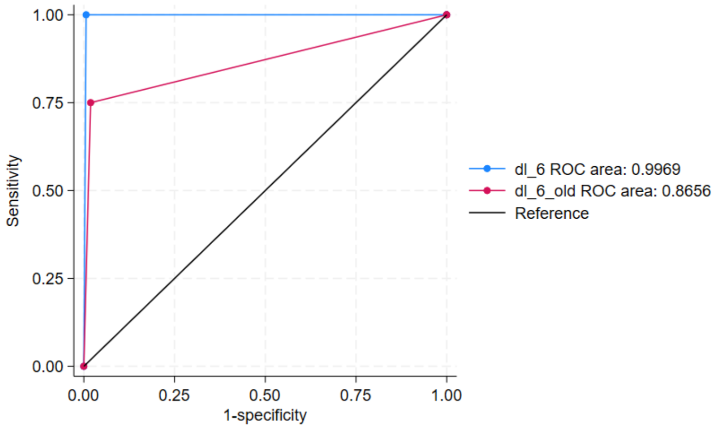

	Obs	ROC area	Std. err.	Asymptotic normal [95% conf.	interval]
dl_6	180	0.9969	0.0031	0.99075	1.00000
dl_6_old	180	0.8656	0.0500	0.76770	0.96355

H0: area(dl_6) = area(dl_6_old)
chi2(1) = 6.93 Prob>chi2 = 0.0085

(g) ROC curves and AUCs on 4808 group (diameter 4.8mm, length 8mm). dl_7: the final DL model, dl_7_old: the DL model with the fine-tuning degree set to zero.

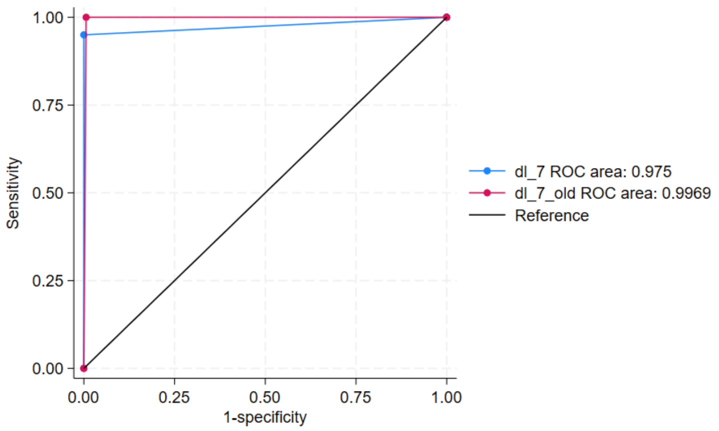

	Obs	ROC area	Std. err.	Asymptotic normal [95% conf.	interval]
dl_7	180	0.9750	0.0250	0.92600	1.00000
dl_7_old	180	0.9969	0.0031	0.99075	1.00000

H0: area(dl_7) = area(dl_7_old)
chi2(1) = 0.75 Prob>chi2 = 0.3853

(h) ROC curves and AUCs on 4810 group (diameter 4.8mm, length 10mm). dl_8: the final DL model, dl_8_old: the DL model with the fine-tuning degree set to zero.

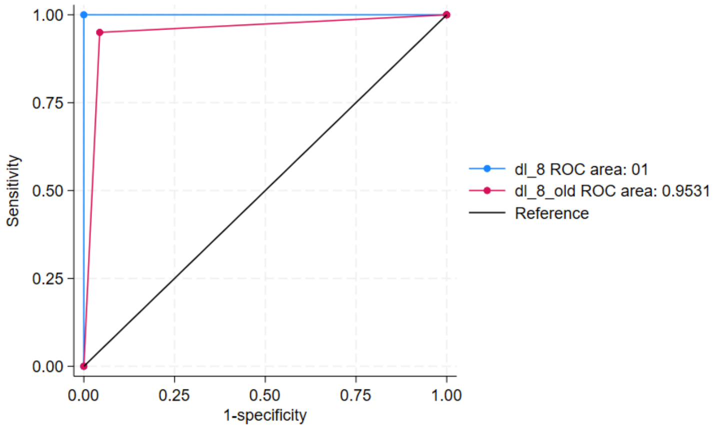

	Obs	ROC area	Std. err.	Asymptotic normal [95% conf.	interval]
dl_8	180	1.0000	0.0000	1.00000	1.00000
dl_8_old	180	0.9531	0.0263	0.90161	1.00000

H0: area(dl_8) = area(dl_8_old)
chi2(1) = 3.18 Prob>chi2 = 0.0745

(i) ROC curves and AUCs on 4812 group (diameter 4.8mm, length 12mm). dl_9: the final DL model, dl_9_old: the DL model with the fine-tuning degree set to zero.

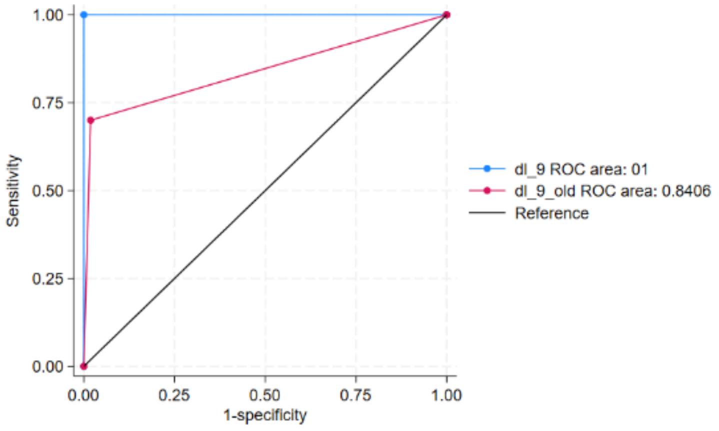

	Obs	ROC area	Std. err.	Asymptotic normal [95% conf.	interval]
dl_9	180	1.0000	0.0000	1.00000	1.00000
dl_9_old	180	0.8406	0.0528	0.73706	0.94419

H0: area(dl_9) = area(dl_9_old)
chi2(1) = 9.10 Prob>chi2 = 0.0026

Supplementary Figure S6. Confusion matrix of the clustering model with the weight of the feature vector set to one.

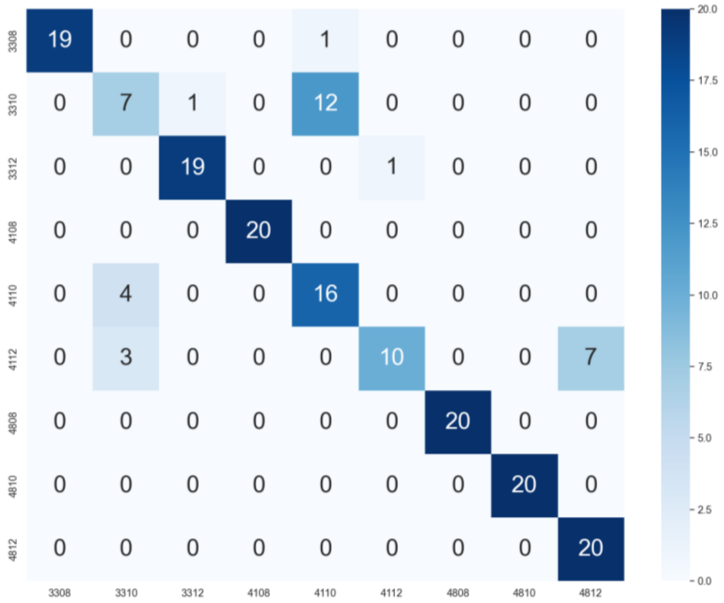

Supplementary Table S7. Performance evaluation results of the clustering model with the weight of the feature vector set to one.

Model	Label	TP	TN	FP	FN	ACC	SE	SP	F1-score	PPV	NPV	AUC-ROC (95% CI)
CL	3308	19	160	0	1	0.994	0.950	1.000	0.974	1.000	0.994	0.975 (0.926-1.000)
	3310	7	153	7	13	0.889	0.350	0.956	0.412	0.500	0.922	*0.653 (0.545-0.762)
	3312	19	159	1	1	0.989	0.950	0.994	0.950	0.950	0.994	0.972 (0.922-1.000)
	4108	20	160	0	0	1.000	1.000	1.000	1.000	1.000	1.000	1.000 (1.000-1.000)
	4110	16	147	13	4	0.906	0.800	0.919	0.653	0.552	0.974	*0.859 (0.767-0.952)
	4112	10	159	1	10	0.939	0.500	0.994	0.645	0.909	0.941	*0.747(0.634-0.859)
	4808	20	160	0	0	1.000	1.000	1.000	1.000	1.000	1.000	1.000 (1.000-1.000)
	4810	20	160	0	0	1.000	1.000	1.000	1.000	1.000	1.000	1.000 (1.000-1.000)
	4812	20	153	7	0	0.961	1.000	0.956	0.851	0.741	1.000	*0.978 (0.962-0.994)

CL, clustering ; TP, true positive; TN, true negative; FP, false positive; FN, false negative; ACC, accuracy; SE, sensitivity; SP, specificity; PPV, positive predictive value; NPV, negative predictive value; AUC, area under the receiver operating characteristic curve; CI, confidence interval. The asterisks (*) indicate statistically significant differences between the model performance before and after tuning, with a significance level of $p < 0.05$.

Supplementary Figure S8. ROC curves and AUC values of the clustering models with the best accuracy and the weight of the feature vector set to one in each of nine groups.

(a) ROC curves and AUCs on 3308 group (diameter 3.3mm, length 8mm). cl_1: the final clustering model, cl_ old_1: the clustering model with the weight of the feature vector set to one.

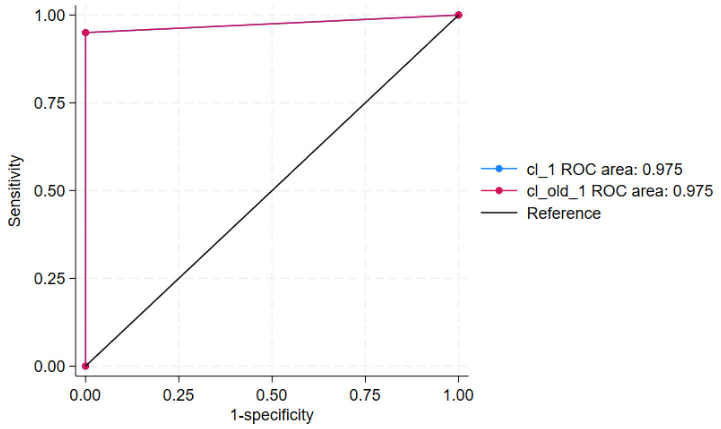

	Obs	ROC area	Std. err.	Asymptotic normal [95% conf.	interval]
cl_1	180	0.9750	0.0250	0.92600	1.00000
cl_old_1	180	0.9750	0.0250	0.92600	1.00000

H0: area(cl_1) = area(cl_old_1)
chi2(0) = 0.00 Prob>chi2 = .

(b) ROC curves and AUCs on 3310 group (diameter 3.3mm, length 10mm). cl_2: the final clustering model, cl_ old_2: the clustering model with the weight of the feature vector set to one.

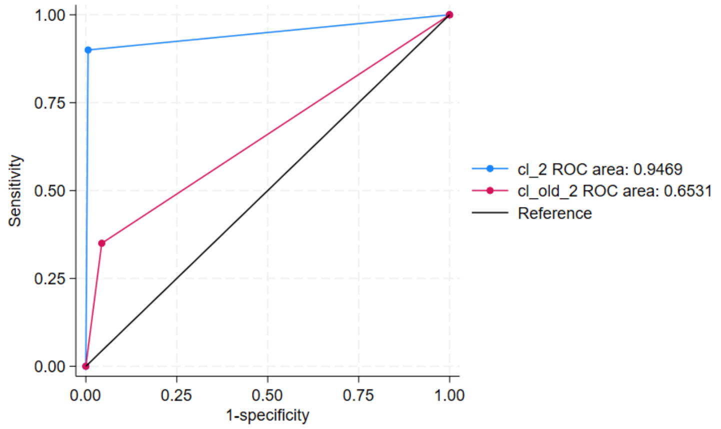

	Obs	ROC area	Std. err.	Asymptotic normal [95% conf.	interval]
cl_2	180	0.9469	0.0346	0.87915	1.00000
cl_old_2	180	0.6531	0.0553	0.54472	0.76153

H0: area(cl_2) = area(cl_old_2)
chi2(1) = 25.89 Prob>chi2 = 0.0000

(c) ROC curves and AUCs on 3312 group (diameter 3.3mm, length 12mm). cl_3: the final clustering model, cl_old _3: the clustering model with the weight of the feature vector set to one.

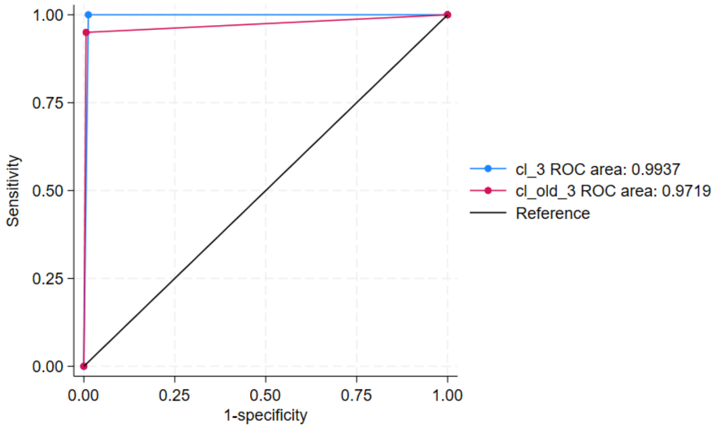

	Obs	ROC area	Std. err.	Asymptotic normal [95% conf. interval]	
cl_3	180	0.9937	0.0044	0.98512	1.00000
cl_old_3	180	0.9719	0.0252	0.92249	1.00000

H0: area(cl_3) = area(cl_old_3)
chi2(1) = 0.75 Prob>chi2 = 0.3853

(d) ROC curves and AUCs on 4108 group (diameter 4.1mm, length 8mm). cl_4: the final clustering model, cl_old _4: the clustering model with the weight of the feature vector set to one.

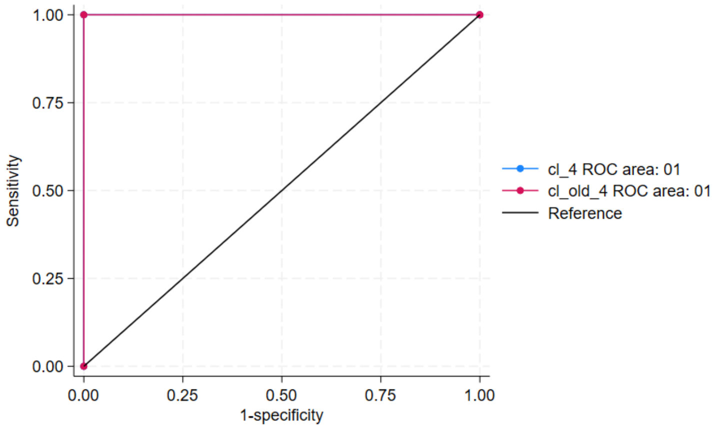

	Obs	ROC area	Std. err.	Asymptotic normal [95% conf. interval]	
cl_4	180	1.0000	0.0000	1.00000	1.00000
cl_old_4	180	1.0000	0.0000	1.00000	1.00000

H0: area(cl_4) = area(cl_old_4)
chi2(0) = 0.00 Prob>chi2 = .

(e) ROC curves and AUCs on 4110 group (diameter 4.1mm, length 10mm). cl_5: the final clustering model, cl_old _5: the clustering model with the weight of the feature vector set to one.

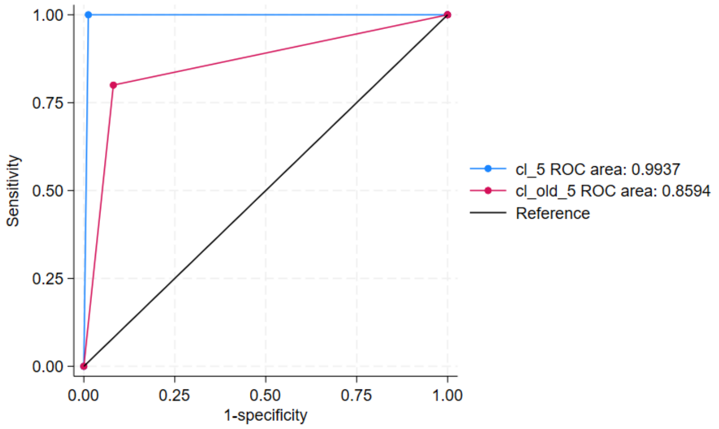

	Obs	ROC area	Std. err.	Asymptotic normal [95% conf. interval]	
cl_5	180	0.9937	0.0044	0.98512	1.00000
cl_old_5	180	0.8594	0.0471	0.76697	0.95178

```
H0: area(cl_5) = area(cl_old_5)
    chi2(1) =      8.04          Prob>chi2 =    0.0046
```

(f) ROC curves and AUCs on 4112 group (diameter 4.1mm, length 12mm). cl_6: the final clustering model, cl_old _6: the clustering model with the weight of the feature vector set to one.

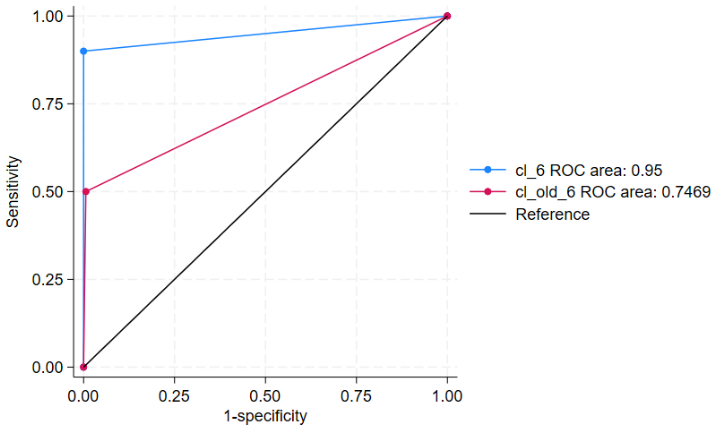

	Obs	ROC area	Std. err.	Asymptotic normal [95% conf. interval]	
cl_6	180	0.9500	0.0344	0.88255	1.00000
cl_old_6	180	0.7469	0.0574	0.63430	0.85945

```
H0: area(cl_6) = area(cl_old_6)
    chi2(1) =     13.03          Prob>chi2 =    0.0003
```

(g) ROC curves and AUCs on 4808 group (diameter 4.8mm, length 8mm). cl_7: the final clustering model, cl_old _7: the clustering model with the weight of the feature vector set to one.

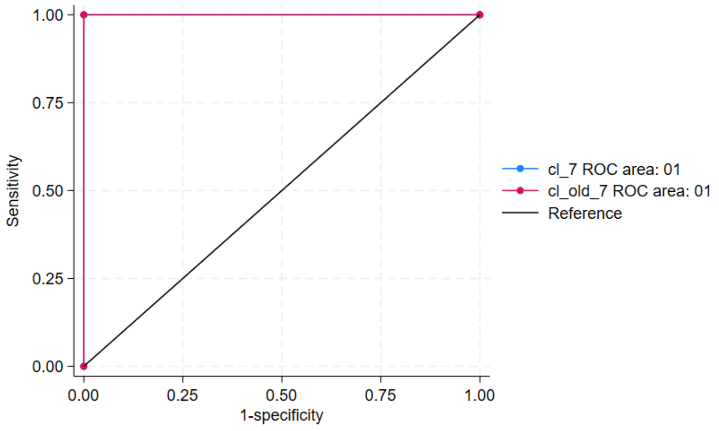

	Obs	ROC area	Std. err.	Asymptotic normal [95% conf.	interval]
cl_7	180	1.0000	0.0000	1.00000	1.00000
cl_old_7	180	1.0000	0.0000	1.00000	1.00000

```
H0: area(cl_7) = area(cl_old_7)
    chi2(0) =      0.00        Prob>chi2 =         .
```

(h) ROC curves and AUCs on 4810 group (diameter 4.8mm, length 10mm). cl_8: the final clustering model, cl_old _8: the clustering model with the weight of the feature vector set to one.

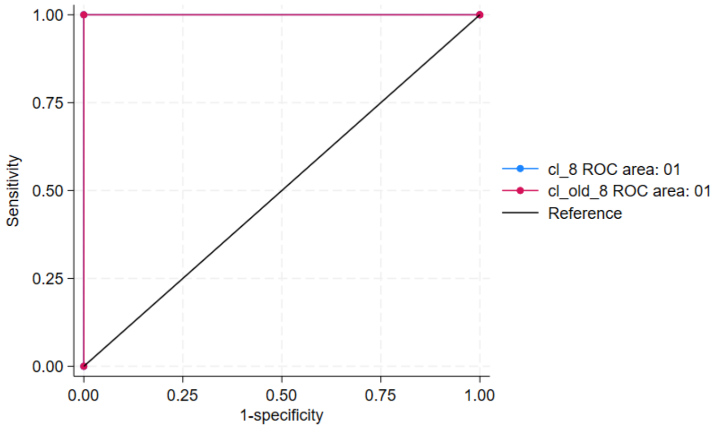

	Obs	ROC area	Std. err.	Asymptotic normal [95% conf.	interval]
cl_8	180	1.0000	0.0000	1.00000	1.00000
cl_old_8	180	1.0000	0.0000	1.00000	1.00000

```
H0: area(cl_8) = area(cl_old_8)
    chi2(0) =      0.00        Prob>chi2 =         .
```

(i) ROC curves and AUCs on 4812 group (diameter 4.8mm, length 12mm). cl_9: the final clustering model, cl_old _9: the clustering model with the weight of the feature vector set to one.

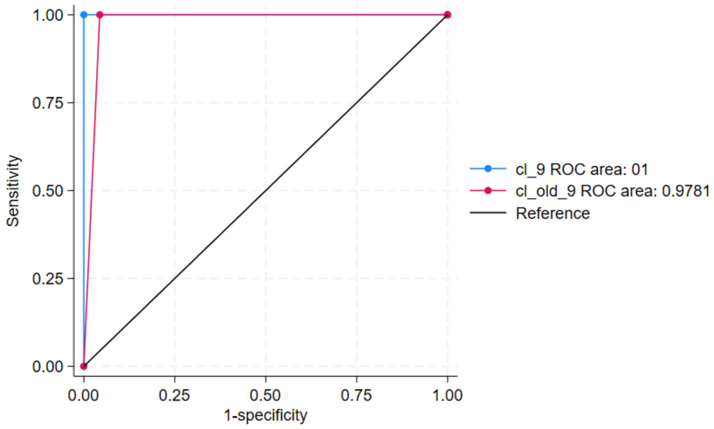

	Obs	ROC area	Std. err.	Asymptotic normal [95% conf.	interval]
cl_9	180	1.0000	0.0000	1.00000	1.00000
cl_old_9	180	0.9781	0.0081	0.96223	0.99402

H0: area(cl_9) = area(cl_old_9)
chi2(1) = 7.27 Prob>chi2 = 0.0070
